# The relationship between hypertension and health-related physical fitness in older adult residences in Wuhan: a cross-sectional study

**DOI:** 10.3389/fpubh.2025.1555638

**Published:** 2025-08-07

**Authors:** Wen Luo, Xi Chen, Jue Wang, Sisi Ke, Wei Zhang, Mei Yang, Yan Guo

**Affiliations:** ^1^Wuhan Centre for Disease Prevention and Control, Wuhan, China; ^2^Maternal and Child Health Hospital of Hongshan District, Wuhan, China; ^3^School of Public Health, Wuhan University of Science and Technology, Wuhan, China

**Keywords:** hypertension, health related physical fitness, aging population, cross-sectional study, Wuhan

## Abstract

**Aim:**

This study aimed to investigate hypertension-associated disparities in health-related physical fitness (HRPF) among community-dwelling older adults in Wuhan.

**Methods:**

This cross-sectional study employed multi-stage stratified random sampling: random selection of 7 districts from Wuhan’s 17 administrative districts and 3–5 communities per district. A total of 801 eligible adults aged ≥65 years were recruited (after exclusions). The sample size was determined using 2015 municipal census data (α = 0.05, power = 90%). Assessments included verification of hypertension and demographic questionnaires, as well as seven standardized HRPF tests across four domains. The data were analyzed using SPSS 13.0, which involved calculating descriptive statistics (means ± SDs, 95% CIs) and performing between-group comparisons (χ^2^ tests and ANOVA).

**Results:**

Hypertensive participants (*n* = 291) demonstrated impaired dynamic balance: they had reduced One-Leg Balance (OLB) time (3.38 ± 3.30 s vs. 3.98 ± 3.81 s; *p* = 0.02). Decreased aerobic endurance: fewer 2-min step repetitions (65.62 ± 29.17 vs. 70.13 ± 26.71; *p* = 0.033). However, they showed enhanced shoulder flexibility: greater back scratch reach (−14.11 ± 12.36 cm vs. -10.47 ± 11.15 cm; *p* < 0.001).

**Conclusion:**

Hypertension is associated with domain-specific HRPF impairments, particularly in balance and aerobic endurance. Targeted exercise interventions that prioritize these domains may mitigate functional decline and reduce cardiovascular burden in aging populations.

## 1 Introduction

China has become an aging society, with 164.5 million citizens aged 65 and above (1). Hypertension is a prevalent condition among the older population in China, affecting 60.56% of individuals aged 65 years and older (2, 3). The burden of hypertension and its complications imposes significant economic and health costs not only on affected individuals but also on their families and society at large (4). Consequently, addressing population aging and reducing hypertension are critical public health priorities in China.

Among diverse strategies for hypertension prevention, physical activity constitutes a core intervention approach. Regular physical activity generally helps prevent hypertension by improving large artery structure and peripheral circulatory function (5). Aerobic exercise modulates hypertension through various pathways, including improvements in insulin sensitivity, regulation of autonomic nervous system function, and reduction of oxidative stress (6, 7). Resistance training primarily influences vasoconstriction regulation (8). These differential mechanisms yield varying clinical effects on blood pressure (9, 10). Therefore, it is essential to establish objective indices for assessing physical activity capacity to optimize outcomes in hypertension prevention.

Health-related physical fitness (HRPF) refers to an individual’s capacity to maintain sufficient energy for daily activities (including occupational and cognitive tasks) without undue fatigue (11, 12). Evidence suggests that exercise modalities have differential effects on HRPF components (13). HRPF includes four core dimensions: muscular strength, aerobic endurance, flexibility, and balance, collectively reflecting integrated physiological function (14–16). Existing literature predominantly indicates that inadequate HRPF is associated with the development of hypertension in older populations (14, 16–18). However, the findings are not studied as a composite whole; research typically focuses on individual dimensions. For instance, studies have shown that good flexibility, muscle strength, or aerobic endurance alone can each reduce the incidence of hypertension (19–21). Conversely, multidimensional studies indicate that compared with aerobic endurance, the effect of muscle strength on hypertension is negligible (19). Conversely, research on children and adolescents is more extensive (19, 22, 23). Overall, existing research lacks a focus on the older population, while HRPF studies have typically been confined to a single dimension (14, 24, 25). We therefore hypothesize that hypertensive older adults in Wuhan exhibit significant HRPF disparities across all domains compared to normotensive peers. To address these gaps, this study aims to examine hypertension-HRPF relationships across all HRPF dimensions.

## 2 Materials and methods

### 2.1 Study design and participants

This cross-sectional study was conducted by the Disease Burden Innovation Research Department at the Wuhan Center for Disease Control and Prevention.

A stratified multistage random sampling design was implemented across Wuhan’s 17 administrative districts. Firstly, seven districts were systematically selected using a random sampling. Secondly, randomly select 3–5 communities per district (a total of 28 communities). Finally, we employed random sampling to enroll adults aged ≥65 years from community registries, targeting 300 participants per district (each community 60–100 subjects, initial N = 2,100). Inclusion criteria: community-dwelling adults aged ≥65 years; provision of written informed consent. Exclusion criteria: any of the following conditions: age <65 years; advanced cardiovascular disease, neuromuscular disorders (e.g., Parkinson’s disease, amyotrophic lateral sclerosis), acute hospitalization (current or within 3 months prior to assessment); cognitive impairment affecting consent capacity.

Field assessments were completed, resulting in 2,150 evaluations across all sites. After database construction and quality control, 54 ineligible samples were excluded, yielding a final analytical sample of 2,096 participants. Subsequently, 1,295 participants were excluded due to non-hypertensive comorbidities affecting fitness outcome (metabolic disorders, type 2 diabetes, dyslipidemia requiring medication, renal impairment, neuromuscular conditions, Parkinson’s disease). The final analytical sample included 801 community-dwelling older adults with isolated hypertension or normotension.

Hypertension was defined as meeting any one of the following criteria: either self-reported physician-diagnosed hypertension by certified healthcare institutions; or current use of antihypertensive medication (self-reported with verification of medication names); or objectively measured elevated blood pressure: Systolic BP ≥ 140 mmHg and/or Diastolic BP ≥ 90 mmHg (based on the average of three seated measurements using calibrated sphygmomanometers after 5-min rest).

### 2.2 General characteristics assessment

General characteristics were assessed using an investigator-developed questionnaire that captured key domains, including demographics (age, sex, marital status, and education level). It also included clinical history of physician-diagnosed chronic conditions (hypertension, diabetes mellitus, stroke, cardiovascular disease, and hyperlipidemia), and addressed lifestyle factors such as physical activity levels and smoking status (current/former/never). To ensure questionnaire quality, the project team implemented standardized investigator training and used uniform procedures and operational protocols for administering surveys to older participants.

### 2.3 HRPF assessment

Seven validated tests were administered to assess four domains of HRPF, following standardized protocols with prioritization of safety (Table 1). Participants were instructed to perform at their natural pace without exertion; incomplete tests were documented without penalty. Each test included 1–2 practice trials before formal recording. Measurement instruments include timing devices: digital stopwatches (mobile phone applications permitted); distance measurement: graduated tape measure (precision: 0.1 cm). Equipment includes: an adjustable-height chair (seat height of 43 cm, backrest secured against the wall); dumbbells (3.6 kg for males, 2.25 kg for females); cones for marker placement; a calibrated sphygmomanometer and weight scale; blood pressure measurement instruments were provisioned by community health centers serving each residential area. The equipment inventory comprised both mercury sphygmomanometers and oscillometric devices across communities. Though heterogeneous in manufacturers and models, all devices underwent rigorous calibration traceable to national metrology institutes.

**Table 1:** HRPF across five domains.

Domain	Test	Protocol
Muscle strength	30-s arm curl test	Seated position, elbow flexion with dumbbell (full extension to 90° shoulder flexion). Count completed repetitions in 30 s (partial final repetition counted).
	30-s chair stand test	Hands crossed over chest, full stands from a seated position. Repetitions counted over 30s (chair stabilized against the wall).
Aerobic endurance	2-min step test	Stationary stepping with knee elevation to mid-patella height. Only bilateral completions are counted. Safety support provided (wall/table).
Flexibility	Chair sit-and-reach test	Legs extended, fingertips measured relative to toes (negative values = shortfall; positive = beyond).
	Back scratch test	Spinal rotation reach distance recorded (negative = gap; positive = overlap)—best of two attempts.
Agility/balance	8-foot timed up-and-go test	Timed rise-walk-return task (distance: 2.5 m). Hand use is permitted during the standing phase.
	One-Leg Balance (OLB)	Eyes-open, non-dominant foot lifted, and timed until balance loss (best of two trials; recorded to 0.1 s).

All assessments were administered by certified community health workers. HRPF measurements were conducted in accordance with the American College of Sports Medicine (ACSM) Guidelines for Exercise Testing and Prescription, 9th Edition (39).

### 2.4 Statistical analysis

Data were analyzed using SPSS 13.0 (IBM Corp), and normality was assessed using the Kolmogorov–Smirnov test. For descriptive statistics, categorical variables are represented as frequencies and percentages, while continuous variables are expressed as mean ± standard deviation (SD) if normally distributed, or as the median and interquartile range if non-normally distributed. Between-group comparisons were conducted using Pearson’s chi-squared test for categorical variables and one-way ANOVA for normally distributed continuous variables. Statistical significance was defined as a two-tailed *p*-value of less than 0.05 (*p* < 0.05) (see Tables 2, 3).

**Table 2:** General characteristics-participation distribution.

Characteristics		Non-hypertensive group	Hypertension group	Asymptotic significance
Sex	Male	164	254	0.074
		56.4%	49.8%	
	Female	127	256	
		43.6%	50.2%	
Age group	65-69Y	117	167	0.194
		40.2%	32.7%	
	70-74Y	90	170	
		30.9%	33.3%	
	75-79Y	50	103	
		17.2%	20.2%	
	≧80Y	34	70	
		11.7%	13.7%	
Smoking statues	Smoking	75	92	0.01
		25.8%	18.0%	
	Non-smoking	216	418	
		74.2%	82.0%	
Physical activity statues	No physical activities	85	176	0.031
		29.2%	34.5%	
	Low-intensity	159	282	
		54.6%	55.3%	
	Moderate-intensity	47	52	
		16.2%	10.2%	

**Table 3:** Comparison of HRPF between hypertension and non-hypertensive groups.

HRPF	Non-hypertensive group	Hypertension group	*p*
OLB	3.98 ± 3.813	3.379 ± 3.3023	0.020
30-s chair stand	13.27 ± 4.82	13.37 ± 4.802	0.777
Chair sit-and-reach	−3.7344 ± 8.42644	−3.5382 ± 8.7238	0.758
30-s arm curl	16.63 ± 8.524	16.05 ± 7.394	0.320
2-min step	70.13 ± 26.706	65.62 ± 29.167	0.033
Back scratch	−10.472 ± 11.1543	−14.111 ± 12.3595	0
8-foot up-and-go	9.537 ± 4.0294	9.446 ± 3.6951	0.747

## 3 Result

### 3.1 General characteristics of participants with hypertension and without hypertension

The analytical sample included 801 community-dwelling older adults (418 males and 383 females), derived from 2096 screened participants after excluding 1,295 individuals with diabetes or comorbidities that could influence HRPF. Participants were stratified into two groups: the hypertensive group (*n* = 291), which included individuals with hypertension but no other chronic conditions (50.2% female, average age of 73.01 years); the non-hypertensive group (*n* = 510) included normotensive individuals without chronic conditions (43.6% female, average age of 72.15 years). No significant age difference was observed between the groups (*p* = 0.074). Hypertension prevalence showed no significant variation across 5-year age strata (65–69, 70–74, 75–79, ≥80 years). Smoking behavior differed significantly (*p* = 0.01), with lower current smoking prevalence in hypertensive (18.0%) versus non-hypertensive participants (25.8%). Physical activity patterns revealed significant disparities (*p* = 0.031). Hypertensive participants reported higher rates of physical inactivity (34.5% vs. 29.2%), lower engagement in moderate-intensity exercise (10.2% vs. 16.2%), and comparable participation in low-intensity activity (55.3% vs. 54.6%).

### 3.2 Comparison of HRPF between hypertension and non-hypertensive groups

Significant between-group differences emerged in three of seven HRPF measures after comprehensive assessment. Hypertensive participants demonstrated reduced balance capacity with OLB time (3.38 ± 3.30 s vs. 3.98 ± 3.81 s; *p* = 0.02); impaired aerobic endurance with poor performance in the 2-min step test (65.62 ± 29.17 vs. 70.13 ± 26.71; *p* = 0.033); enhanced shoulder flexibility with greater reach in the back scratch test (−14.11 ± 12.36 cm vs-10.47 ± 11.15 cm; *p* < 0.001).

No significant differences were observed in: lower-body strength (30-s chair stand: 13.37 ± 4.80 vs. 13.27 ± 4.82; *p* = 0.777); hamstring flexibility (chair sit-and-reach: −3.54 ± 8.72 cm vs. -3.73 ± 8.43 cm; *p* = 0.758); upper-body strength (30-s arm curl: 16.05 ± 7.39 vs. 16.63 ± 8.52; *p* = 0.320); mobility (8-foot up-and-go: 9.45 ± 3.70 s vs. 9.54 ± 4.03 s; *p* = 0.747).

## 4 Discussion

This large-scale cross-sectional study examined hypertension-related disparities in HRPF among community-dwelling older adults in Wuhan. Our key findings reveal complex relationships between hypertension status and functional capacity, with implications for the development of targeted interventions.

Consistent with global trends, we observed no significant differences in hypertension prevalence across age groups, marital status, or education levels (1). However, hypertensive participants demonstrated significantly lower smoking rates (18.0% vs. 25.8%; *p* = 0.01), aligning with evidence that a hypertension diagnosis motivates smoking cessation (26). This behavioral shift may stem from heightened health awareness, as hypertensive smokers face elevated risks of malignant hypertension and accelerated atherosclerosis (27). Notably, both groups exhibited higher smoking rates than European and African cohorts, suggesting region-specific public health challenges (26, 28). Beside these, hypertensive older adults showed significantly higher inactivity rates (34.5% vs. 29.2%) and lower moderate-intensity exercise participation (10.2% vs. 16.2%). This activity profile may directly contribute to the observed HRPF deficits.

Within the specific HRPF domain, our study identified three significant differences. Hypertensive participants demonstrated significantly shorter OLB times (*p* = 0.020) and reduced performance on the 2-min step test (*p* = 0.033), indicating impaired balance and diminished aerobic endurance. Conversely, enhanced back scratch reach (*p* < 0.001) reflected superior flexibility in the hypertensive group. The aerobic endurance deficit likely indicates impaired oxygen delivery due to vascular endothelial dysfunction. Disrupted nitric oxide (NO) bioavailability, the principal endothelium-derived relaxing factor (EDRF) that mediates vascular smooth muscle relaxation, elevates peripheral resistance, and reduces skeletal muscle perfusion (29–31). Research in both humans and rodents has demonstrated that elevated nitric oxide levels improve endothelium-dependent vasodilation (32, 33). In patients with essential hypertension, the impairment of endothelial function and regulation of vascular tone results in increased peripheral vascular resistance and inadequate modulation of oxygen delivery to skeletal muscle, thereby affecting muscle function. Muscular function impairment may also help explain the impaired balance capacity in the hypertensive group (30).

Our study also demonstrated better flexibility in the hypertensive group. This finding contrasts with an earlier report that found a persistent inverse association between greater flexibility and lower hypertension incidence after controlling for confounders (34). While this observed flexibility advantage needs further investigation, it may indicate compensatory adaptation to balance limitations. Additionally, the potential confounding influence of sex cannot be excluded, particularly given the overrepresentation of females (who typically exhibit enhanced flexibility) in the hypertensive group (35).

Our results reinforce specific HRPF indexes associated with hypertension (17, 18, 36). Targeted exercise interventions should focus on aerobic training to improve endothelial function and oxygen delivery (37). Such interventions may reduce the increased frailty risk observed in hypertensive older adults (38).

## 5 Conclusion

This cross-sectional study demonstrated significant disparities in HRPF between hypertensive and non-hypertensive community-dwelling older adults in Wuhan. Key findings indicate that hypertensive older adults exhibit impaired functional capacities. Accelerated age-related decline is characterized by faster deterioration in aerobic endurance, flexibility, and dynamic balance compared to non-hypertensive peers. Activity pattern differences include higher rates of physical inactivity (34.5% vs. 29.2%) and lower engagement in moderate-intensity exercise.

These functional limitations likely originate from hypertension-associated vascular pathophysiology and suboptimal activity profiles. Implementing targeted exercise interventions, particularly combining aerobic training with balance-challenging components, may mitigate functional decline, reduce cardiovascular risk, and alleviate the growing disease burden affecting both families and society.

## 6 Limitations

This study has several limitations. Design limitations include the cross-sectional methodology, which precludes causal inference between HRPF and the development of hypertension. Unmeasured confounders, such as potential influencing factors (e.g., sex-specific fitness patterns, nutritional status, and medication adherence), were not fully evaluated. Activity assessment, including physical activity levels, depended on self-report without accelerometer validation. Future research should address these issues through prospective cohort studies with serial HRPF measurements. Furthermore, mechanistic investigations of hypertension-related neuromuscular decline and randomized controlled trials testing tailored exercise regimens for hypertensive older adults are recommended.

## Data Availability

The raw data supporting the conclusions of this article will be made available by the authors, without undue reservation.

## References

[1] Fang EF Xie C Schenkel JA Wu C Long Q Cui H. A research agenda for ageing in China in the 21st century (2nd edition): focusing on basic and translational research, long-term care, policy and social networks. Ageing Res Rev. (2020) 64:101174. doi: 10.1016/j.arr.2020.101174, PMID: 32971255 PMC7505078

[2] Chaoyang Z Gang Z Juan D Jie G. Prevalence, awareness and control rates of hypertension among elderly aged ≥65 years in Wuhan. Chin J Hypertens. (2014) 22:5.

[3] Zhang W Yan Y Liu X Zhou X Guo Y. Impact of hypertension, diabetes, hyperlipidemia and their comorbidities on cognitive function in elderly. Chin Prev Med. (2021) 22:411–7. doi: 10.16506/j.1009-6639.2021.06.003

[4] Qiu Y Ma J Zhu J Liu Y Ren W Zhang S. Deaths and disability-adjusted life years of hypertension in China, South Korea, and Japan: a trend over the past 29 years. Front Cardiovasc Med. (2023) 10:1080682. doi: 10.3389/fcvm.2023.1080682, PMID: 37008311 PMC10050598

[5] De Ciuceis C Rizzoni D Palatini P. Microcirculation and physical exercise in hypertension. Hypertension. (2023) 80:730–9. doi: 10.1161/HYPERTENSIONAHA.122.19465, PMID: 36601920

[6] Moraes-Silva IC Mostarda C Moreira ED Silva KA dos Santos F de Angelis K. Preventive role of exercise training in autonomic, hemodynamic, and metabolic parameters in rats under high risk of metabolic syndrome development. J Appl Physiol. (2013) 114:786–91. doi: 10.1152/japplphysiol.00586.2012, PMID: 23329818

[7] Korsager Larsen M Matchkov VV. Hypertension and physical exercise: the role of oxidative stress. Medicina (Kaunas). (2016) 52:19–27. doi: 10.1016/j.medici.2016.01.005, PMID: 26987496

[8] Araujo AJ Santos AC Souza KD Aires MB Santana-Filho VJ Fioretto ET. Resistance training controls arterial blood pressure in rats with L-NAME-induced hypertension. Arq Bras Cardiol. (2013) 100:339–46. 23545992

[9] Rossi AM Moullec G Lavoie KL Gour-Provencal G Bacon SL. The evolution of a Canadian hypertension education program recommendation: the impact of resistance training on resting blood pressure in adults as an example. Can J Cardiol. (2013) 29:622–7. doi: 10.1016/j.cjca.2013.02.010, PMID: 23541664

[10] Börjesson M Onerup A Lundqvist S Dahlöf B. Physical activity and exercise lower blood pressure in individuals with hypertension: narrative review of 27 RCTs. Br J Sports Med. (2016) 50:356–61. doi: 10.1136/bjsports-2015-095786, PMID: 26787705

[11] Boutcher YN Boutcher SH. Exercise intensity and hypertension: what's new? J Hum Hypertens. (2017) 31:157–64. doi: 10.1038/jhh.2016.62, PMID: 27604656

[12] Tremblay MS Colley RC Saunders TJ Healy GN Owen N. Physiological and health implications of a sedentary lifestyle. Appl Physiol Nutr Metab. (2010) 35:725–40. doi: 10.1139/H10-079, PMID: 21164543

[13] Dong B Beauchamp M Hao Q. Exercise for sarcopenia in older people: a systematic review and network meta-analysis. J Cachexia Sarcopenia Muscle. (2023) 14:1199–211. doi: 10.1002/jcsm.13225, PMID: 37057640 PMC10235889

[14] Yumei L Zuoliang W Yunhong W Yaoxiu C Enping L. Health-related physical fitness and chronic diseases analysis in active elderly adults. Chin J Geriatr. (2015) 34:561–4. doi: 10.3760/cma.j.issn.0254-9026.2015.05.028

[15] Rikli RE Jones CJ. Development and validation of criterion-referenced clinically relevant fitness standards for maintaining physical independence in later years. Gerontologist. (2013) 53:255–67. doi: 10.1093/geront/gns071, PMID: 22613940

[16] Ho CC Lee PF Chen HL Tseng CY Hsieh XY Chiu CH. Poor health-related physical fitness performance increases the overweight and obesity risk in older adults from Taiwan. BMC Geriatr. (2021) 21:170. doi: 10.1186/s12877-021-02112-1, PMID: 33750323 PMC7941954

[17] Navarrete-Villanueva D Gómez-Cabello A Marín-Puyalto J Moreno LA Vicente-Rodríguez G Casajús JA. Frailty and physical fitness in elderly people: a systematic review and meta-analysis. Sports Med. (2021) 51:143–60. doi: 10.1007/s40279-020-01361-1, PMID: 33201455

[18] Faselis C Doumas M Kokkinos JP Panagiotakos D Kheirbek R Sheriff HM. Exercise capacity and progression from prehypertension to hypertension. Hypertension. (2012) 60:333–8. doi: 10.1161/HYPERTENSIONAHA.112.196493, PMID: 22753224

[19] Crump C Sundquist J Winkleby MA Sundquist K. Interactive effects of physical fitness and body mass index on the risk of hypertension. JAMA Intern Med. (2016) 176:210–6. doi: 10.1001/jamainternmed.2015.7444, PMID: 26784837 PMC4803286

[20] Nishiwaki M Kurobe K Kiuchi A Nakamura T Matsumoto N. Sex differences in flexibility-arterial stiffness relationship and its application for diagnosis of arterial stiffening: a cross-sectional observational study. PLoS One. (2014) 9:e113646. doi: 10.1371/journal.pone.0113646, PMID: 25427157 PMC4245207

[21] Luo JH Zhang TM Yang LL Cai YY Yang Y. Association between relative muscle strength and hypertension in middle-aged and older Chinese adults. BMC Public Health. (2023) 23:2087. doi: 10.1186/s12889-023-17007-6, PMID: 37880652 PMC10598916

[22] Ortega FB Ruiz JR Castillo MJ Sjostrom M. Physical fitness in childhood and adolescence: a powerful marker of health. Int J Obes. (2008) 32:1–11. doi: 10.1038/sj.ijo.0803774, PMID: 18043605

[23] Agostinis-Sobrinho C Ruiz JR Moreira C Lopes L Ramírez-Vélez R García-Hermoso A. Changes in muscular fitness and its association with blood pressure in adolescents. Eur J Pediatr. (2018) 177:1101–9. doi: 10.1007/s00431-018-3164-4, PMID: 29740692

[24] Leong DP Teo KK Rangarajan S Lopez-Jaramillo P Avezum A Jr Orlandini A. Prognostic value of grip strength: findings from the prospective urban rural epidemiology (PURE) study. Lancet. (2015) 386:266–73. doi: 10.1016/S0140-6736(14)62000-6 25982160

[25] Cohen DD Gómez-Arbeláez D Camacho PA Pinzon S Hormiga C Trejos-Suarez J. Low muscle strength is associated with metabolic risk factors in Colombian children: the ACFIES study. PLoS One. (2014) 9:e93150. doi: 10.1371/journal.pone.0093150, PMID: 24714401 PMC3979680

[26] Bager JE Mourtzinis G Andersson T Nåtman J Rosengren A Björck S. Trends in blood pressure, blood lipids, and smoking from 259 753 patients with hypertension in a Swedish primary care register: results from QregPV. Eur J Prev Cardiol. (2022) 29:158–66. doi: 10.1093/eurjpc/zwab087 34056646

[27] Virdis A Giannarelli C Neves MF Taddei S Ghiadoni L. Cigarette smoking and hypertension. Curr Pharm Des. (2010) 16:2518–25. doi: 10.2174/138161210792062920, PMID: 20550499

[28] Noubiap JJ Nansseu JR Endomba FT Ngouo A Nkeck JR Nyaga UF. Active smoking among people with diabetes mellitus or hypertension in Africa: a systematic review and meta-analysis. Sci Rep. (2019) 9:588. doi: 10.1038/s41598-018-37858-z, PMID: 30679752 PMC6345945

[29] Caspersen CJ Powell KE Christenson GM. Physical activity, exercise, and physical fitness: definitions and distinctions for health-related research. Public Health Rep. (1985) 100:126–31. PMID: 3920711 PMC1424733

[30] Nyberg M Gliemann L Hellsten Y. Vascular function in health, hypertension, and diabetes: effect of physical activity on skeletal muscle microcirculation. Scand J Med Sci Sports. (2015) 25:60–73. doi: 10.1111/sms.12591, PMID: 26589119

[31] Das S Kumar KN. Nitric oxide: its identity and role in blood pressure control. Life Sci. (1995) 57:1547–56. doi: 10.1016/0024-3205(95)02130-b, PMID: 7564902

[32] Hotta K Behnke BJ Arjmandi B Ghosh P Chen B Brooks R. Daily muscle stretching enhances blood flow, endothelial function, capillarity, vascular volume and connectivity in aged skeletal muscle. J Physiol. (2018) 596:1903–17. doi: 10.1113/JP275459, PMID: 29623692 PMC5978284

[33] Bisconti AV Cè E Longo S Venturelli M Coratella G Limonta E. Evidence for improved systemic and local vascular function after long-term passive static stretching training of the musculoskeletal system. J Physiol. (2020) 598:3645–66. doi: 10.1113/JP279866, PMID: 32613634

[34] Gando Y Sawada SS Momma H Kawakami R Miyachi M Lee IM. Body flexibility and incident hypertension: the Niigata wellness study. Scand J Med Sci Sports. (2021) 31:702–9. doi: 10.1111/sms.13867, PMID: 33141990

[35] Araújo CGS de Souza E Silva CG Kunutsor SK Franklin BA Laukkanen JA Myers J. Reduced body flexibility is associated with poor survival in middle-aged men and women: a prospective cohort study. Scand J Med Sci Sports. (2024) 34:e14708. doi: 10.1111/sms.14708, PMID: 39165228

[36] Lopez-Jaramillo P Lopez-Lopez JP Tole MC Cohen DD. Muscular strength in risk factors for cardiovascular disease and mortality: a narrative review. Anatol J Cardiol. (2022) 26:598–607. doi: 10.5152/AnatolJCardiol.2022.1586, PMID: 35924286 PMC9403882

[37] Kim YH Jeong MK Park H Park SK. Effects of regular taekwondo intervention on health-related physical fitness, cardiovascular disease risk factors and epicardial adipose tissue in elderly women with hypertension. Int J Environ Res Public Health. (2021) 18:2935. doi: 10.3390/ijerph18062935, PMID: 33809392 PMC7999820

[38] Yang Y Xu H Liu X Li J Liew Z Liu X. Joint association of smoking and physical activity with mortality in elderly hypertensive patients: a Chinese population-based cohort study in 2007-2018. Front Public Health. (2022) 10:1005260. doi: 10.3389/fpubh.2022.1005260, PMID: 36249230 PMC9558130

[39] Thompson PD Arena R Riebe D Pescatello LS American College of Sports Medicine. ACSM'S new preparticipation health screening recommendations from ACSM'S guidelines for exercise testing and prescription, ninth edition. Curr Sports Med Rep. (2013) 12:215–7. doi: 10.1249/JSR.0b013e31829a68cf, PMID: 23851406

